# Optic neuritis as the initial clinical presentation of limbic encephalitis: a case report

**DOI:** 10.1186/s13256-018-1893-7

**Published:** 2018-12-03

**Authors:** Stephanie S. L. Cheung, Gary K. K. Lau, Koon-Ho Chan, Ian Y. H. Wong, Jimmy S. M. Lai, Wai Kiu Tang, Kendrick C. Shih

**Affiliations:** 1 Department of Ophthalmology and Visual Sciences, Hong Kong Eye Hospital, 147K Argyle Street, Kowloon, Hong Kong SAR; 2 Department of Medicine, Li Ka Shing Faculty of Medicine, 21 Sassoon Road, Pokfulam, Hong Kong SAR; 3 Department of Ophthalmology, Li Ka Shing Faculty of Medicine, University of Hong Kong, 301B Cyberport 4, 100 Cyberport Road, Pokfulam, Hong Kong SAR; 4 Department of Imaging and Interventional Radiology, The Chinese University of Hong Kong, Prince of Wales Hospital, 30-32 Ngan Shing Street, Shatin, Hong Kong SAR

**Keywords:** Optic neuritis, Limbic encephalitis, Autoimmune encephalitis, Paraneoplastic syndrome

## Abstract

**Background:**

Limbic encephalitis is characterized by rapid onset of working memory deficit, mood changes, and often seizures. The condition has a strong paraneoplastic association, but not all cases are invariably due to tumors.

**Case presentation:**

We present a case of limbic encephalitis in a Chinese patient who initially presented to our hospital with optic neuritis and no other neurological symptoms. The diagnosis was made radiologically, and cognitive and neurological symptoms did not occur until 5 months later. Extensive investigations for autoimmune, infective, and neoplastic causes were all negative. A working diagnosis of paraneoplastic neurological syndrome was made, and the patient is being managed with high-dose steroid therapy according to the Optic Neuritis Treatment Trial protocol during relapses, as well as with tumor surveillance.

**Conclusions:**

This case highlights ocular symptoms as important clues for diagnosing neurological diseases, as well as autoimmune encephalitis as an important differential diagnosis in the management of “idiopathic” optic neuritis in the Chinese population.

## Background

Limbic encephalitis refers to an autoimmune inflammatory process localized to the limbic system. Patients present with cognitive impairment along with disordered perception, mood changes, and sleep disturbances. Limbic encephalitis has a strong paraneoplastic association, but not all cases are invariably due to tumors [1]. A careful history and examination may show early clues to specific autoimmune disease, but a diagnosis is difficult owing to the substantial overlap of symptoms among different types of autoimmune encephalitis, as well as with infective, metabolic, and toxic causes of encephalitis [2, 3]. Tremendous advances in autoimmune encephalitis research in the past 10 years led to the discovery of new syndromes and biomarkers that have changed the diagnostic approach to this disease entity. Specifically, antibody status is not needed to consider limbic encephalitis as having a definite autoimmune origin [4].

## Case presentation

A 42-year-old Chinese woman with good past health presented to our hospital on March 26, 2013, with bilateral progressive blurring of vision for approximately 8 days. She had no redness or photophobia, nor did she have pain at rest or upon eye movement. She did not experience headache, nausea, or vomiting or have any recent history of head trauma. Furthermore, she had no recent flulike illness, upper respiratory tract symptoms, fever, chills, or rigor. Physical examination revealed visual acuity (VA) down to hand movements only on the right eye and finger-counting on the left, as well as bilateral red color desaturation. Pupillary light reflexes were present but sluggish, with a right relative afferent pupillary defect detected. Extraocular movements were full, and intraocular pressure was normal in both eyes. Slit-lamp examination revealed normal anterior segments with no evidence of inflammation. Dilated fundal examination showed pink discs with sharp margins and cup-to-disc ratios of 0.3 bilaterally. Bilateral retinas were normal with dry maculas. Neurological examination revealed intact cranial nerves, normal limb power and sensation, generalized brisk but symmetrical jerks with downgoing plantar reflexes, and absence of cerebellar signs. No changes in sensorium or psychotic features were noted.

**Fig. 1:**
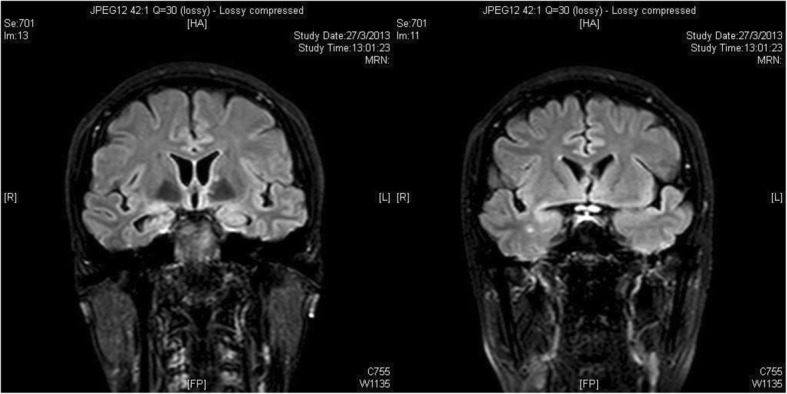
Magnetic resonance imaging of the brain with contrast performed on 27 March 2013. Abnormal T2-weighted (T2W) hyperintense signal is seen along the bilateral amygdala and hippocampi, resembling limbic encephalitis. Bilateral optic nerves are enlarged, exhibiting T2W hyperintensity with contrast enhancement compatible with optic neuritis

Magnetic resonance imaging (MRI) of the brain with contrast the next day revealed radiological evidence of bilateral optic neuritis (ON), limbic and cortical encephalitis, subcortical and pontine myelitis, and leptomeningitis (Fig. 1). Blood tests revealed normal white blood cell counts, liver and renal function, and inflammatory markers, including C-reactive protein and erythrocyte sedimentation rate. The patient’s Venereal Disease Research Laboratory (VDRL) test result was nonreactive, and her test result for Lyme disease was negative. An extensive panel of autoimmune antibodies was negative, including antinuclear antibody, anti-double-stranded DNA antibody, anti-extractable nuclear antigen, and rheumatoid factor. Results of testing for specific antibodies for autoimmune encephalitis were also negative, including anti-aquaporin 4 antibody, anti-myelin oligodendrocyte glycoprotein, anti-voltage-gated potassium channel antibody, and anti-*N*-methyl-d-aspartate receptor antibodies. Antibodies against paraneoplastic antigens were screened, including anti-neuronal nuclear antibody type I, anti-neuronal nuclear antibody type II (anti-Ma2), anti-CRMP5 antibody, anti-Yo, anti-Ri, and anti-Hu, and all results were negative. Tumor markers, including cancer antigens CA 15-3 and CA 19-9 as well as carcinoembryonic antigen, and α-fetoprotein were all within normal limits. A lumbar puncture was performed, showing normal opening pressure of 12 mmHg, normal cerebrospinal fluid (CSF) cell count and biochemistry, and absence of oligoclonal proteins, as well as negative microbiological findings for bacterial, mycobacterial, and fungal growth; cryptococcal antigen; herpes simplex virus; and *Mycobacterium tuberculosis* complex PCR; and VDRL. Positron emission tomography-computed tomography (PET-CT) revealed no hypermetabolic lesion or tumor focus. Optical coherence tomography of the nerve fiber layer showed bilateral nasal thinning, consistent with myopic eyes with tilted disc configuration. In view of a likely underlying autoimmune etiology, a negative lumbar puncture microbiological workup, and evidence of limbic involvement found upon neuroimaging, a presumptive diagnosis of autoimmune limbic encephalitis was made. The patient was started on high-dose pulsed steroid therapy (intravenous methylprednisolone 1 g/day over four divided doses) 3 days after admission for 72 hours, followed by switching to oral steroids with gradual tapering according to the Optic Neuritis Treatment Trial protocol [1]. Over the next few months, the patient’s VA and color vision slowly improved from hand movement to 0.7 bilaterally.

**Fig. 2:**
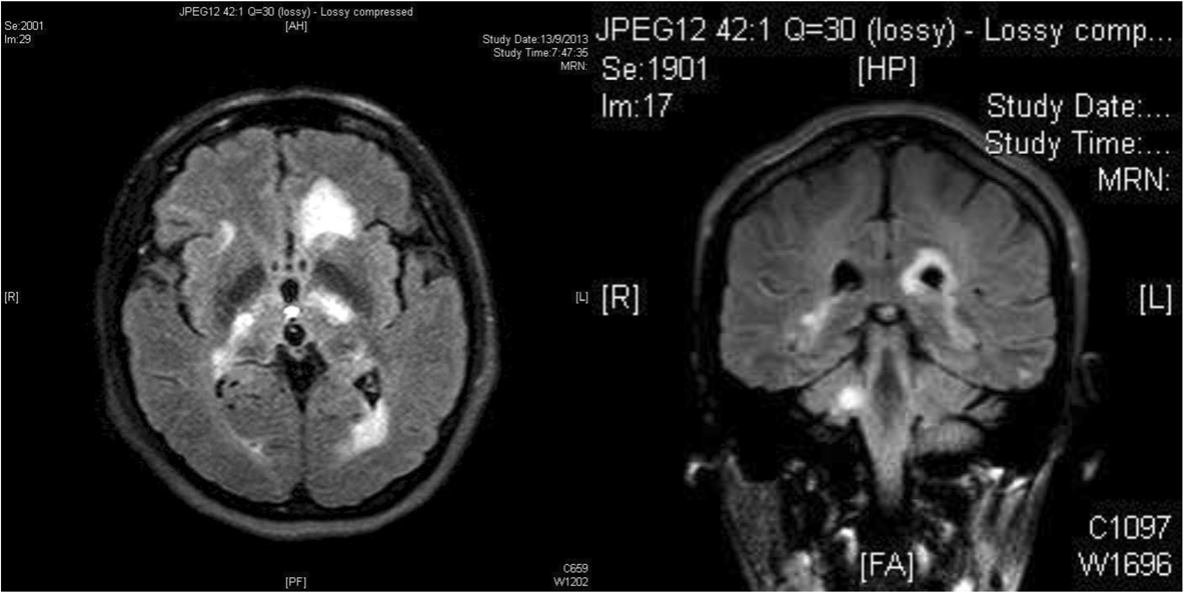
Magnetic resonance imaging of the brain with contrast performed on 13 September 2013. New periventricular and subcortical white matter of bilateral cerebral hemispheres, left-sided corpus callosum, left thalamus, left brainstem, and right cerebellar peduncle

MRI of the cervical and thoracic spine, with and without contrast, in July 2013 showed no evidence of myelitis or demyelinating lesions. On follow-up in August 2013, however, the patient reported cognitive slowing, new-onset facial asymmetry, slurring of speech, right upper limb weakness, and unsteady gait. Physical examination revealed right upper limb power 4/5, brisk reflexes throughout, and right lower limb ataxia. The patient’s VA had decreased to 20/200 bilaterally, with persistent sluggish pupil response. Repeat MRI of the brain with contrast in September 2013 showed resolution of previous white matter lesions but new periventricular and subcortical white matter of bilateral cerebral hemispheres, left-sided corpus callosum, bilateral internal capsules, left thalamus, bilateral hippocampal tails, left brainstem, and right cerebellar peduncle (Fig. 2). Lumbar puncture and whole-body PET-CT were repeated, which showed no abnormalities. In view of relapse, the patient was restarted on intravenous methylprednisolone, followed by maintenance therapy with oral prednisolone with the aim of tapering the steroid over time. She was also started on azathioprine 25 mg/day beginning 30 September 2013, with the aim of increasing the dosage over time in order to replace oral prednisolone.

VA improved back to 0.7 bilaterally after initiation of immunosuppressant therapy. Limb power returned to full strength by November 2013. Throughout the next 2 years, the patient had slow improvement of VA back to 0.7 bilaterally and color vision recovering bilaterally. She was tapered off oral steroids by March 2016, and she was found to have stable VA at her last follow-up in July 2016. She is currently being followed with annual PET-CT imaging for tumor surveillance as well as annual serological surveillance.

## Discussion

ON refers to the inflammation of the optic nerve associated with visual disturbances and characteristic optic nerve dysfunction. In its most typical form, ON is an inflammatory demyelinating process that is most associated with multiple sclerosis (MS) in the Caucasian population. In atypical forms, ON is associated with non-MS immune-mediated causes such as neuromyelitis optica and with systemic disorders such as connective tissue diseases and infective conditions. In our locality, optic perineuritis (OPN), which is a form of idiopathic orbital inflammatory disease specifically affecting the optic nerve sheath and has a presentation similar to that of ON, must be considered as an important differential diagnosis. The diagnosis of OPN is made on the basis of a finding of enhancement around rather than within the optic nerve on an MRI scan. OPN is more commonly encountered in Hong Kong Chinese patients than in Caucasians, and neuroimaging and further investigations are needed to differentiate it from ON, because prompt initiation of steroid therapy was found to be essential for preventing irreversible visual loss and recurrent attacks, whereas the same treatment does not affect final visual outcomes for those with ON [5]. Moreover, although MS is the most common diagnosis associated with ON in the Caucasian population, idiopathic ON is found to be the commonest etiology in Asians [5, 6].

Although MS is a less common etiology of ON in the Asian population, some patients without the diagnosis of MS at ON presentation do develop MS during subsequent follow-up [5–8]. Recent reports of concurrent emergence of autoimmune encephalitis and MS [9–12] highlight the increasing recognition of ON as a presenting symptom in autoimmune encephalitis, with or without association with a demyelinating disorder. Autoimmune encephalitis was not a well-known clinical entity until recent advances in immunology gave way to the discovery of novel forms of encephalitis associated with antibodies to cell surface or synaptic proteins. However, a recent landmark paper on the clinical approach to diagnosis of autoimmune encephalitis de-emphasized the reliance on antibody testing and instead placed importance on clinical assessment and conventional tests that are accessible to most clinicians, because an absence of antibodies does not exclude the possibility of an immune-mediated etiology and a positive test does not always imply an accurate diagnosis [4].

On the basis of the proposed diagnostic criteria of definite autoimmune limbic encephalitis (Table 1), our patient could not be unequivocally labeled as having a case of limbic encephalitis, because she had no overt psychiatric symptoms, nor did her CSF demonstrate pleocytosis. Electroencephalography was not performed at the time, and she had a negative test result for anti-Hu and anti-Ma2, two antibodies that are predominantly associated with limbic encephalitis. In other words, the diagnosis of limbic encephalitis was made radiologically. The decision to manage this patient on the basis of a presumptive diagnosis of limbic encephalitis was made because of the observation that 60–70% of paraneoplastic autoimmune encephalitis cases present with neurological symptoms before the detection of underlying cancer [2]; as such, despite our patient’s atypical presentation of limbic encephalitis, we believed it was prudent to closely monitor her with regular tumor surveillance, given the definite radiological evidence of limbic involvement. Finally, as the disease progresses over time, it is possible to detect antibodies that were not previously present, which would lend definitive support to the diagnosis of limbic encephalitis.

**Table 1:** Diagnostic criteria of limbic encephalitis

Diagnosis of limbic encephalitis can be made when all four^a^ of the following criteria have been met:	
1. Subacute onset (rapid progression of less than 3 months) of working memory deficits, seizures, or psychiatric symptoms suggesting involvement of the limbic system	
2. Bilateral brain abnormalities on T2-weighted fluid-attenuated inversion recovery MRI highly restricted to the medial temporal lobes	
3. At least one of the following:	
• CSF pleocytosis (white blood cell count of more than 5 cells/mm^3^)	
• EEG with epileptic or slow-wave activity involving the temporal lobes	
4. Reasonable exclusion of alternative causes	

*Abbreviations: CSF* Cerebrospinal fluid, *EEG* Electroencephalography, *MRI* Magnetic resonance imaging

Adapted from Graus *et al*. [4]

^a^If one of the first three criteria is not met, a diagnosis of definite limbic encephalitis can be made only with the detection of antibodies against cell surface, synaptic, or onconeural proteins

## Conclusions

We present a case of a patient with ON with a presumptive etiology of limbic encephalitis. This case report highlights (1) limbic encephalitis as a differential diagnosis in considering Chinese patients with “idiopathic” ON (that is, those who do not fulfill the diagnostic criteria of MS or other well-recognized demyelinating diseases such as neuromyelitis optica); (2) the importance of imaging in differentiating ON and OPN, because the latter requires prompt steroid therapy to avoid significant rapid visual deterioration; (3) the recent departure from relying on detection of autoantibodies for the diagnosis of autoimmune limbic encephalitis; and (4) the need for tumor surveillance in patients with suspected autoimmune limbic encephalitis.

## Abbreviations

Anti-Ma2: Anti-neuronal nuclear antibody type II
CSF: Cerebrospinal fluid
EEG: Electroencephalography
MRI: Magnetic resonance imaging
MS: Multiple sclerosis
ON: Optic neuritis
OPN: Optic perineuritis
PET-CT: Positron emission tomography-computed tomography
VA: Visual acuity
VDRL: Venereal Disease Research Laboratory
